# Explain Bioinformatics to Your Grandmother!

**DOI:** 10.1371/journal.pcbi.1003305

**Published:** 2013-10-31

**Authors:** Virginie Bernard, Magali Michaut

**Affiliations:** 1 Next Generation Sequencing Platform - ICGex, Curie Institute, Paris, France; 2 Computational Cancer Biology, The Netherlands Cancer Institute, Amsterdam, The Netherlands; Princeton University, United States of America

## Abstract

What are you working on? You have certainly been asked that question many times, whether it be at a Saturday night party, during a discussion with your neighbors, or at a family gathering. Communicating with a lay audience about scientific subjects and making them attractive is a difficult task. But difficult or not, you will have to do it for many years, not only with your family and friends, but also with your colleagues and collaborators. So, better learn now! Although not usually taught, the ability to explain your work to others is an essential skill in science, where communication plays a key role. Using some examples of the French Regional Student Group activities, we discuss here (i) why it is important to have such communication skills, (ii) how you can get involved in these activities by using existing resources or working with people who have previous experience, and (iii) what you get out of this amazing experience. We aim to motivate you and provide you with tips and ideas to get involved in promoting scientific activities while getting all the benefits.

## Presenting Research to a Lay Audience Is Important

Being asked what you work on is sometimes a difficult question to answer for a scientist. You should not avoid it but should try to explain clearly and enthusiastically the beauty of your research topic! Communicating with a lay audience is indeed a difficult exercise [1]. What is a lay audience? This could be anyone who is not familiar with your research topic or not accustomed to science discussion, or people generally curious about science, or questioning children. In other words, everyone outside your research laboratory. For them, you should explain the concepts from the start, without assuming preexisting knowledge. In a more professional context, you may have discussions with scientists working in other fields, or even in your field but with different backgrounds and experiences. This is the broad context of popularization: explaining your science to a lay audience. There are several types of events where scientists can talk about their work: scientific open discussions in a cafe, public seminars, specific animation events for a national scientific day, an open day at the research institute, or even when you present your group for potential internship candidates. It is very important to effectively communicate with this audience. Why is it so essential? For many reasons, including the global education and integration of science into society and the recruitment of young scientists to build the next generation of researchers. In many countries, science is funded mostly by taxpayers, and it is fair that they know how their money is spent and understand the current progress and challenges in research. Many people believe that science is complicated and that they can not understand it. But that is not true. As a scientist, you can help by clearly explaining the main questions and why they are important for everybody, and by highlighting why these questions are challenging and exciting. This should help citizens to realize that the scientific community is accessible to anybody. In addition, explaining your work and your research field in general is important to create interest for young people, which can lead to a new vocation. The next generation is the future of research. By showing passion for your work, you may kindle interest in others and nurture it by mentoring them. This can be true for young children, adolescents, and even other scientists discovering a new field of research. The inspiration of a mentor is often a key point in a scientific career [2].

## Young Researchers Have a Role to Play

Even though this communication is important and useful at any stage in your career, as a young scientist, you can bring something specific to it. Indeed young scientists are often very dynamic and passionate about a field they have newly discovered. Young audiences can feel closer to young scientists and realize such speakers are accessible and a welcome departure from the stereotype of a crazy scientist in a lab coat. In addition, young scientists typically have less responsibility and more time to get involved in spreading this message than senior scientists. Organizing events is time consuming so you had best have time to do it well. Young scientists are a good bridge between the experts and a lay audience. That is why the Student Council of the International Society for Computational Biology and its Regional Student Groups are getting involved in such activities. We will illustrate some concepts in this article with activities led by the French Regional Student Group (RSG-France).

When you have the opportunity to explain your projects or your work, an important point in engaging your audience is to avoid the technical jargon of your field. We have all been in discussions with friends who used abbreviations nobody but experts were able to understand. How boring! In order to understand, we had to ask lots of questions. This is not the strategy you should use to talk about your work with a lay audience. Try to use concrete examples to highlight how fun and fascinating your work is. RSG-France used these approaches when promoting science during scientific days for the public and in collaboration with another association. These activities are reported below.

## Examples of Activities Presenting Science

First, with the collaboration of local organizers of scientific days for a lay audience, RSG-France developed an interactive game suitable for visitors 7 to 99 years old. The game introduced the concept of DNA sequences and sequences comparison. The public faced an investigation: given a DNA sample—from faeces found in the mountains—could they resolve who it came from? Was it a wolf? A dog? From where did it come? The format of this investigation and the associated story engaged the interest of most visitors. They were guided during the whole game to be as good an investigator as possible, *i.e.*, as good a computational biologist as possible. Moreover, the story told was a real one. Is there a better way to really involve people than having a real-life game? At the end, participants who completed the investigation—and all of them did—were rewarded with statements of accomplishment from the research lab, similar to what a student or a young researcher might receive from a teacher after graduating. This game has been used for three years by local collaborators and is always a great success. People enjoy playing and do not realize they are learning about the work of a researcher in bioinformatics. The funnier the activity, the easier to learn.

As a second scientific popularization activity, RSG-France worked in collaboration with a company bringing students to enjoy a scientific holiday, a summer camp. Roundtables were organized with students and researchers sharing their passions in a fun way. Informal talks and discussions make the young students more amenable to understanding the field. In this context, RSG-France organized a bioinformatics workshop for teenagers. The goal was to describe bioinformatics to young people and foster vocations. The main fields of bioinformatics were introduced. With theoretical and mainly practical sessions, the workshop helped attendees to initiate—or improve—their bioinformatics knowledge. The work done was useful for anybody wanting to introduce what working in bioinformatics means.

## Tips on Presenting Science

A key point in effectively explaining your job and speaking about one particular field to a lay audience is to keep it simple. While scientists are used to reading detailed research reports for information that relates to their own project, nonexperts wishing to understand what science is about are not familiar with such material. Do not try to exhaustively explain the story of bioinformatics or the details of your current research project with your key questions and hypotheses. Only a few people in your laboratory or in your field will be interested. And if your grandmother told you she was interested, it is just because she enjoys talking with you. You can be sure she did not remember a word of your talk! Do your best to deliver knowledge: explain science with comparisons to familiar examples from the everyday world. For example, genes are book chapters and amino acids are words. All together, genes tell a story. Transcription and translation are reading a book from start to end and understanding it. A genome is a collection of books telling a big story, the story of someone's life. What about bioinformatics? It is the only way to analyze the whole story! To read all the books one by one would be extremely time consuming. Therefore the use of a computer is required. Computational biologists use computers to help understand the story of our lives. Remember that you are more likely to get people's attention if you do not aim to teach. In front of a nonexpert community, be sure to make your talk enjoyable. Having fun and using real examples helps your audience to remember things. Remember, people learn much better from a talk or activity when they enjoy it.

If you are a beginner in scientific popularization, you might consider contributing to an existing initiative. More generally you can use books [3] and web resources, and draw on the skills and experience of local and national associations involved in such activities, colleagues, and friends. Professors may be used to popularizing science with new students. Some associations are dedicated to such activities. They have the experience you need. You can—and should—ask them for advice. They will enjoy helping you. They may have some slides, a story, or a website you could use. Why not reuse work already done, as RSG-France did? There is no benefit in reinventing things when you can apply existing ones to new purposes. Once ready for scientific popularization, enjoy talking to the public. You will meet people highly interested in what you are presenting. Their interest may be evident in their enthusiasm or in their questions on the topic. It is extremely rewarding for speakers to feel this attraction to science related to their work.

## Conclusion

Science is an important part of society and it is one of the roles of scientists to communicate about their work with the lay audience. It is even useful for you, as, according to Einstein, “You do not really understand something unless you can explain it to your grandmother.” In addition, communicating about your research with your colleagues is part of your daily work. Any collaboration would be more efficient with better communication.

**About the Authors**

The authors have been involved in many aspects of the ISCB Student Council and specifically worked together in the French Regional Student Group (RSG-France, JeBiF). **Virginie Bernard** was secretary (2010–2011) of the RSG-France and served on its Board of Directors from 2009 until 2012. **Magali Michaut** was co-founder and president (2008–2010) of RSG-France and served on its Board of Directors from 2008 until now. She was co-founder, secretary (2009), and president (2010–2011) of RSG-Europe. In addition, she served as secretary for the ISCB Student Council in 2009.
